# Supplementary material for: The ISW1 and CHD1 chromatin remodelers suppress global nucleosome dynamics in living yeast cells
Source: bioRxiv. 2025 Apr 23:2025.04.17.649351. Preprint. [Version 1] doi: 10.1101/2025.04.17.649351 (PMC12190399; doi:10.1101/2025.04.17.649351)
Supplement: 1 [file NIHPP2025.04.17.649351v1-supplement-1.pdf]

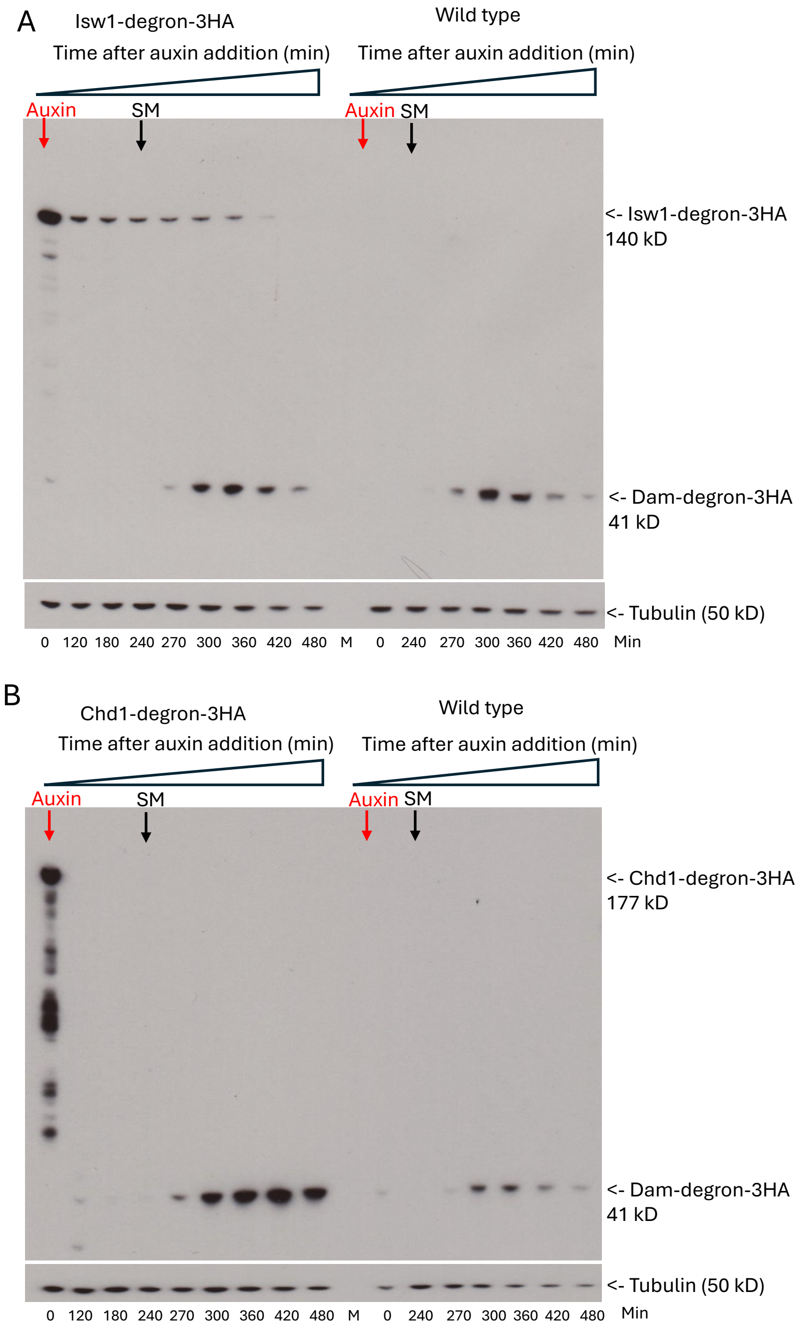

Fig. S1. Chd1-depleted cells produce more Dam than wild type cells.

(A) Anti-HA immunoblot performed to follow Isw1 depletion and Dam induction. Wild type and Isw1–degron cells were treated with auxin for 4 h and then induced with SM for another 4 h. Our analysis begins with the 240 min time point, when SM was added (= 0 min). (**B**) Anti-HA immunoblot showing higher Dam induction compared to wild type after depletion of Chd1.

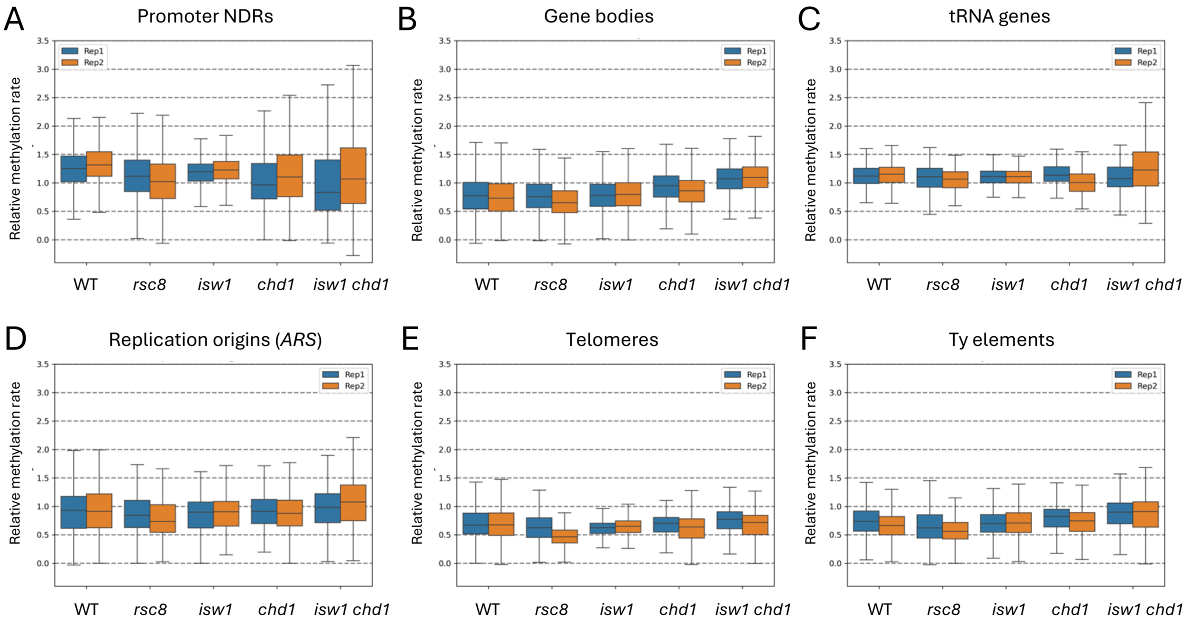

Fig. S2. Comparison of normalized average Dam methylation rates in vivo in various genomic elements: wild type vs. cells depleted of remodeler subunits. The methylation rate was calculated for each GATC site in the yeast genome and normalized to the internal median mtDNA methylation rate (set to 1). (**A**) Promoter NDRs. (**B**) Gene bodies. (**C**) tRNA genes. (**D**) Replication origins (*ARS* elements). (**E**) Telomeres. (**F**) Ty transposable elements. The box contains 25%-75% of the data; the line is the median; the whiskers represent 1.5 times the inter-quartile range to the farthest data points.

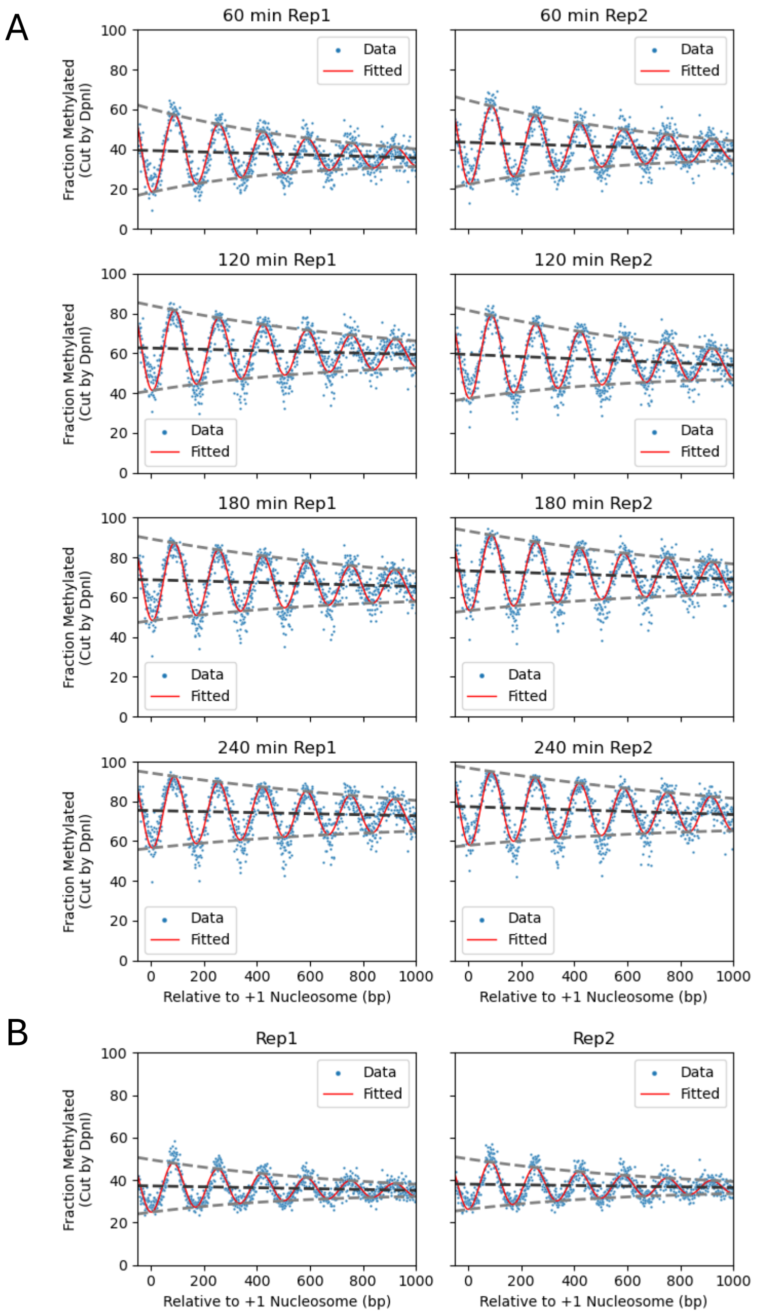

Fig. S3. Decaying sine wave model fit to Dam methylation data for wild type, remodeler-depleted cells and for isolated nuclei. (**A**) Sine wave model fit to time course data for wild type cells. The methylated fraction was calculated for each GATC site in the yeast genome at each time point after SM addition. The methylated fraction is plotted relative to the location of the +1 nucleosome dyad of 5398 genes. Values for individual GATC sites are indicated by blue dots. The decaying sine wave fit is shown as a red line. The baseline of the sine wave is indicated by the dark grey dashed line; the decaying amplitudes are indicated by light grey dashed lines. (**B**) Analysis of limit methylation data for wild type nuclei treated with 5.6 nM Dam.

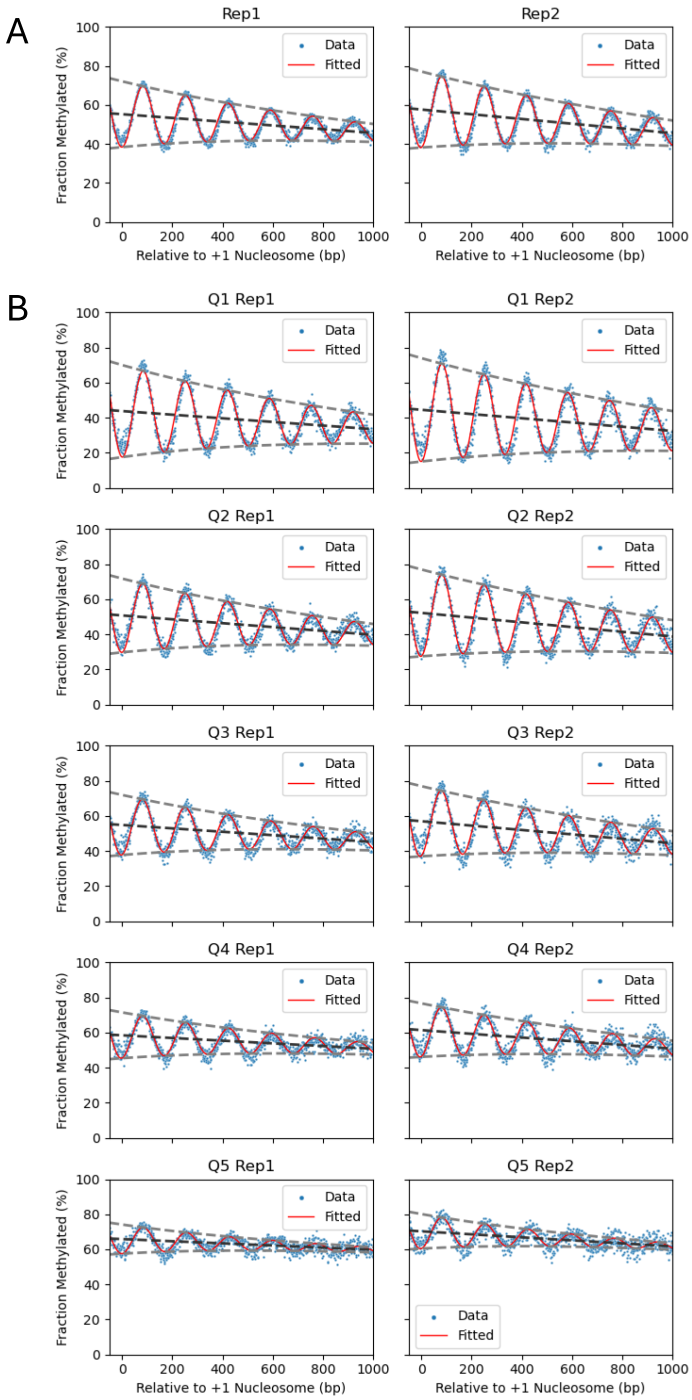

Fig. S4. Decaying sine wave model fit to M.SssI methylation nanopore data for wild type cells induced with SM for 240 min in vivo. (**A**) Analysis of all genes. The methylated fraction was calculated for each CG site in the yeast genome. Individual CG sites are indicated by blue dots. The decaying sine wave fit is shown as a red line. The baseline of the sine wave is indicated by the dark grey dashed line; the decaying amplitudes are indicated by light grey dashed lines. (**B**) Analysis of genes sorted into quintiles using their calculated individual unbiased average methylation rates. Quintile 1 (Q1) contains the slowest methylating genes.

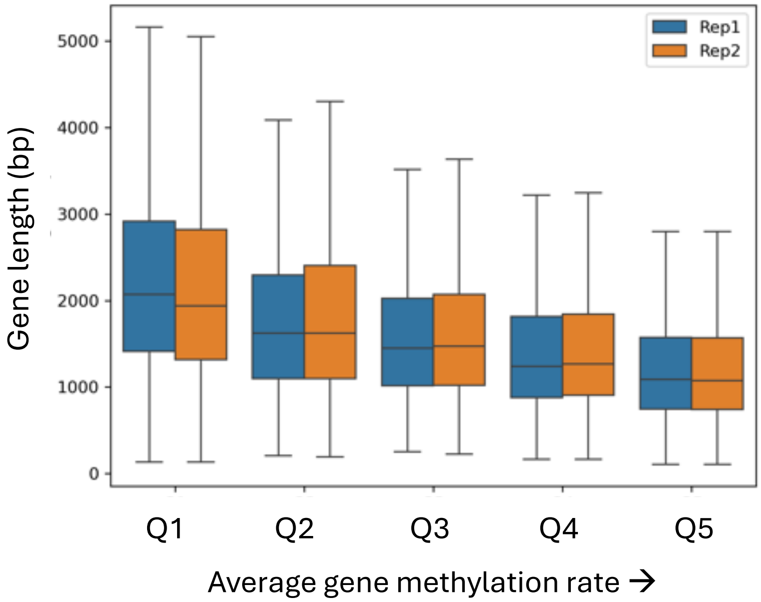

Fig. S5. Gene length distributions in the mean relative M.SssI methylation rate quintiles. The 5398 genes were divided into quintiles according to their individual mean methylation rates, as shown in Fig. 4B. Quintile 5 (Q5) contains the fastest methylating genes. Box plots show the distribution of gene lengths (TSS to TTS) for the genes in each quintile. The box contains 25%-75% of the data; the line is the median; the whiskers represent 1.5 times the inter-quartile range to the farthest data points).

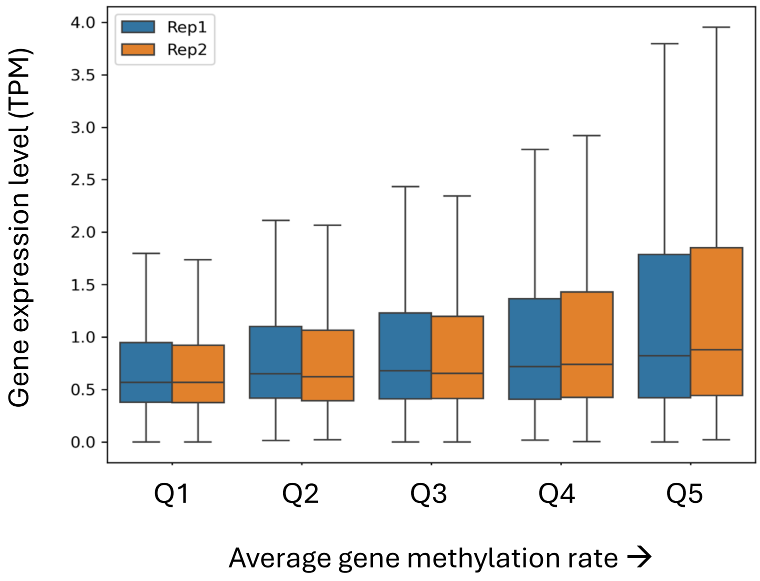

Fig. S6. Transcript level distributions in the mean relative M.SssI methylation rate quintiles. The 5398 genes were divided into quintiles according to their individual mean methylation rates, as shown in Fig. 4B. Quintile 5 (Q5) contains the fastest methylating genes. Box plots show the distribution of transcript levels for the genes in each quintile (RNA-seq data for SM-induced cells (*33*)) for the genes in each quintile. The box contains 25%-75% of the data; the line is the median; the whiskers represent 1.5 times the inter-quartile range to the farthest data points).

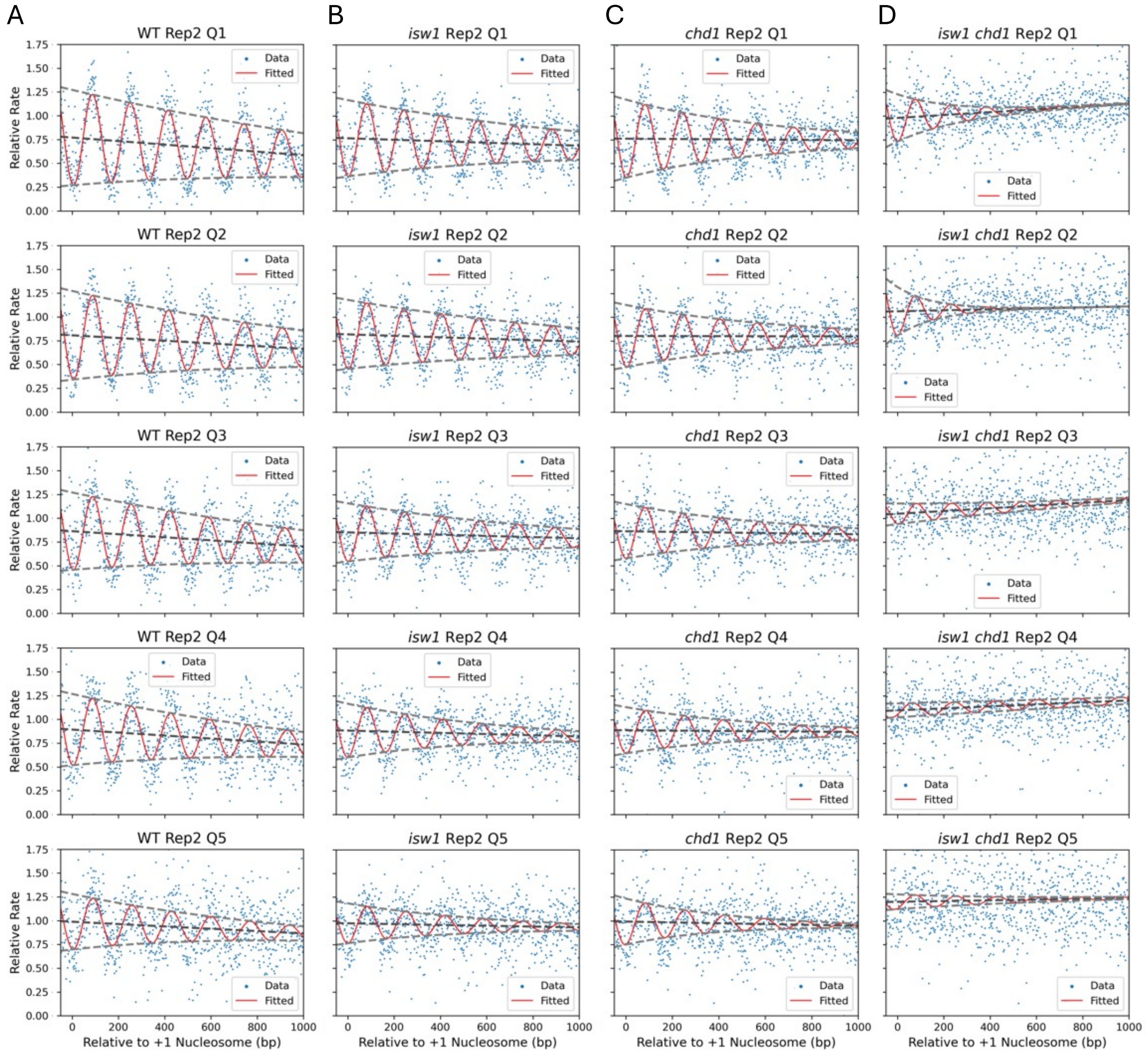

Fig. S7. Decaying sine wave model fit to Dam methylation data for wild type and remodeler-depleted cells in vivo: Comparison of methylation rate quintiles for biological replicate 2. (**A**) wild type; (**B**) Isw1-degron mutant; (**C**) Chd1-degron mutant; (**D**) Isw1-degron and Chd1-degron mutant. (see Table S2 for fit parameters). Dam methylation rate data normalised to mtDNA (set to 1) are plotted for each quintile of genes defined using M.SssI nanopore data for replicate 1 (Fig. 4B). Values for individual GATC sites are indicated by blue dots. The methylation rates for each set of genes are plotted relative to the location of the +1 nucleosome dyad. The decaying sine wave fit is shown as a red line. The baseline of the sine wave is indicated by the dark grey dashed line; the decaying amplitudes are indicated by light grey dashed lines. Data for biological replicate 1 are shown in Fig. 5.

					ln(1 - methylated fraction) for median GATC site				
**Experiment**	**Replicate**	**Rate**	**Intercept**	R^2	**30 min**	**60 min**	**120 min**	**180 min**	**240 min**
WT	1	0.0079	0.114	0.983	-0.110	-0.301	-0.977	-1.224	-1.778
WT	2	0.0085	0.091	0.996	-0.172	-0.402	-0.912	-1.520	-1.915
rsc8	1	0.0055	-0.066	0.985	-0.210	-0.408	-0.699	-1.151	-1.330
rsc8	2	0.0062	-0.121	0.998	-0.334	-0.467	-0.867	-1.210	-1.625
isw1	1	0.0095	0.027	0.982	-0.177	-0.549	-1.161	-1.830	-2.114
isw1	2	0.0089	-0.018	0.973	-0.179	-0.542	-1.223	-1.737	-2.009
chd1	1	0.0142	-0.247	0.907	-0.340	-1.055	-2.452	-3.076	-3.240
chd1	2	0.0148	-0.231	0.922	-0.443	-1.004	-2.386	-3.280	-3.354
isw1 chd1	1	0.0132	-0.330	0.866	-0.366	-1.033	-2.580	-2.924	-3.083
isw1 chd1	2	0.0151	-0.445	0.899	-0.477	-1.367	-2.874	-3.358	-3.664

Table S1. mtDNA methylation rates in vivo for wild type and remodeler depletion mutants. Calculation of 'ln(1 - methylated fraction)' at times after SM addition. The data for wild type (WT) mtDNA replicate 1 are plotted in Fig. 1C. The median methylation rate for the GATC sites in mtDNA is given in the 'Rate' column. The rate is given by the slope of the plot of 'ln(1 - methylated fraction)' vs. time after SM addition (the intercept and the coefficient of determination (R^2) are also provided; formula 2). The distributions of the rate values for individual GATC sites in mtDNA for each replicate are shown in the box plots in Fig. 1D.

Strain	Quintile	Spacing (bp)	Amplitude	Slope per kb	Decay per Period	Adjusted Mean Rate	Adjusted R^2
WT	1	163.9	0.50	-0.18	0.88	0.67	0.66
WT	2	164.2	0.47	-0.14	0.86	0.73	0.58
WT	3	166.4	0.41	-0.16	0.87	0.78	0.47
WT	4	169.0	0.37	-0.15	0.84	0.81	0.36
WT	5	170.3	0.29	-0.12	0.80	0.93	0.22
isw1	1	159.9	0.40	-0.08	0.85	0.72	0.54
isw1	2	160.6	0.36	-0.08	0.86	0.77	0.50
isw1	3	162.7	0.31	-0.06	0.83	0.82	0.36
isw1	4	164.9	0.28	-0.06	0.77	0.85	0.25
isw1	5	167.8	0.21	-0.04	0.75	0.95	0.14
chd1	1	160.0	0.41	-0.02	0.78	0.75	0.42
chd1	2	160.4	0.33	0.00	0.79	0.79	0.32
chd1	3	164.3	0.29	-0.03	0.78	0.84	0.24
chd1	4	168.2	0.24	-0.01	0.76	0.88	0.16
chd1	5	171.8	0.24	-0.05	0.67	0.97	0.09
isw1 chd1	1	-	0.25	+0.15	0.55	1.05	0.12
isw1 chd1	2	-	0.26	+0.05	0.39	1.08	0.07
isw1 chd1	3	-	0.11	+0.15	0.80	1.12	0.05
isw1 chd1	4	-	0.07	+0.11	0.88	1.15	0.02
isw1 chd1	5	-	0.08	+0.04	0.72	1.22	0.00

Table S2. Decaying sine wave model parameters fitted to Dam data for wild type and depletion mutants in vivo. For the quintile data in Fig. S7 (Replicate 2): individual genes grouped by methylation rate (Q5 contains the fastest methylating genes). Parameters are as described in the legend to Table 1. Spacing values are not provided for the Isw1/Chd1 double depletion mutant because the fit is too poor. See Table 3 for the fit to biological replicate 1.
